# Effects of Hypoxia and Transferrin on Toxicity and DNA Binding of Ruthenium Antitumor Agents in Hela Cells

**DOI:** 10.1155/MBD.1996.197

**Published:** 1996

**Authors:** D. Frasca, J. Ciampa, J. Emerson, R. S. Umans, M. J. Clarke

**Affiliations:** 1 Merkert Chemistry Center Boston College Chestnut Hill MA 02167 USA

## Abstract

Nuclear DNA binding and inhibition of growth of HeLa cells in culture were determined after 24 h incubation with the ruthenium anticancer agents *cis*-[Cl_2_(NH_3_)_4_Ru]Cl (CCR) and (ImH)*trans*-[(Im)_2_Cl_4_Ru] (ICR) as a function of [Ru], Po_2_, and added transferrin. Consistent with the “activation-by-reduction” hypothesis, cytotoxicity and DNA binding for both complexes increased under reduced oxygen conditions. Consistent with the “transferrin- transport” hypothesis, inhibition of cell growth also increased with added transferrin for both complexes. Despite their differences in charge, reduction potentials and substitution rates, both complexes behaved remarkably similarly indicating a common mechanism of action for both. Under atmospheric Conditions (Po_2_ = 159 torr), CCR inhibited HeLa cell growth with IC_50_ = 3.5 μM, while that for ICR was 2.0 μM. The binding of both complexes to DNA (Ru_DNA_/P_DNA_) correlated with toxicity and was approximately linear in the concentration of the ruthenium complex in the culture medium, [Ru]. For both complexes, IC_50_ values decrease and DNA binding increases with decreasing log(Po_2_). In general, DNA binding at all oxygen pressures for both complexes is in the range of one Ru per 1000-2000 DNA base pairs at [Ru] = IC_50_.


### EFFECTS OF HYPOXIA AND TRANSFERRIN ON TOXICITY AND DNA BINDING OF RUTHENIUM ANTITUMOR AGENTS IN HELA CELLS

D. Frasca, J. Ciampa, J. Emerson, R. S. Umans, and M. J. Clarke*

Merkert Chemistry Center, Boston College, Chestnut Hill, MA 02167, USA

**Abstract:** Nuclear DNA binding and inhibition of growth of HeLa cells in culture were determined after 24 h incubation with the ruthenium anticancer agents *cis*-$[Cl_2(NH_3)_4Ru]Cl$ (CCR) and (ImH)*trans*-$[(Im)_2Cl_4Ru]$ (ICR) as a function of [Ru], $P_{O_2}$, and added transferrin. Consistent with the "activation-by-reduction" hypothesis, cytotoxicity and DNA binding for both complexes increased under reduced oxygen conditions. Consistent with the "transferrin-transport" hypothesis, inhibition of cell growth also increased with added transferrin for both complexes. Despite their differences in charge, reduction potentials and substitution rates, both complexes behaved remarkably similarly indicating a common mechanism of action for both. Under atmospheric conditions ($P_{O_2}$ = 159 torr), CCR inhibited HeLa cell growth with $IC_{50}$ = 3.5 µM, while that for ICR was 2.0 µM. The binding of both complexes to DNA ($Ru_{DNA}/P_{DNA}$) correlated with toxicity and was approximately linear in the concentration of the ruthenium complex in the culture medium, [Ru]. For both complexes, $IC_{50}$ values decrease and DNA binding increases with decreasing $\log(P_{O_2})$. In general, DNA binding at all oxygen pressures for both complexes is in the range of one Ru per 1000-2000 DNA base pairs at [Ru] = $IC_{50}$.

### Introduction

A number of ammine, amine and heterocyclic complexes of ruthenium exhibit: inhibition of DNA replication,[1,2] mutagenic activity, induction of the SOS repair mechanism,[3] binding to nuclear DNA,[4] and reduction of RNA synthesis,[5] so that DNA is strongly indicated as the target molecule for ruthenium anticancer complexes.

While even some monoacido complexes such as $[CH_3CH_2COO(NH_3)_5Ru^{III}]ClO_4$ exhibit good activity, generally multichloro compounds such as *cis*-$[Cl_2(NH_3)_4Ru]Cl$,*fac*-$[Cl_3(NH_3)_3Ru]$,[1,6] and Na *trans*-$[(Im)_2Cl_4Ru]$[7] exhibit the best activity. In the case of the latter compound, Keppler improved upon the activity of the poorly-soluble $[Cl_3(NH_3)_3Ru]$ by charging with fewer nitrogen ligands and more halides to yield a more soluble anionic complex.[8-10]

An early working hypothesis regarding the design of Ru-anticancer agents suggested a tumor selectivity based upon preferential reduction of $Ru^{III}$-prodrugs in the more reducing environment of tumors.[11,12] The $Ru^{II}$ oxidation state should also be more prevalent inside of tumors, because in the hypoxic milieu of tumors there is less oxygen to oxidize $Ru^{II}$ back to the $Ru^{III}$ state.[13,14] Such "activation by reduction" in the tumor would favor intracellular binding by the more rapidly substituting $Ru^{II}$ ion. Since unprotonated imines are a preferred binding site for $Ru^{II}$, the concentration of such sites in the N7 of purines in nucleic acids suggests DNA as a preferred target.

Srivastava showed that some ruthenium complexes, such as $RuCl_3$, were transported to the tumor by transferrin.[15] Clarke suggested that multiacido ruthenium(III) complexes, particularly anionic or neutral complexes with lower ligand field stabilization energies and more rapid substitution rates, might also be transported in the blood by transferrin.[16-19] Keppler and coworkers have since demonstrated that Na*trans*-$[(Im)_2Cl_4Ru]$[20] and related complexes are indeed bound by apotransferrin in a reversible fashion such that the *trans*-$[(Im)_2Ru]^{3+}$ core remains intact.[18,19] It has been suggested that transferrin binding by this type of complex follows the hydrolytic loss of a chloride ligand and may be specifically mediated by the protein.[21] This result suggests that apotransferrin can act as a natural and somewhat selective carrier of $Ru^{III}$-drugs into tumor cells owing to the large number of transferrin receptors on the their surface.[19]

While it has been implied that ruthenium complexes may operate either by the transferrin-transport or activation-by-reduction mechanisms, these two mechanisms are not mutually exclusive. In this study, we correlate cytotoxicity and DNA binding in human cervical cancer cells (HeLa) with oxygen tension and added apotransferrin concentration. Two active antitumor ruthenium(III) compounds were chosen for investigation. The first, *cis*-$[Cl_2(NH_3)_4Ru]Cl$ (CCR), yields the cationic species, *cis*-$[Cl_2(NH_3)_4Ru]^+$, in solution and was considered more likely to function by the activation-by-reduction route, while the second, [ImH]*trans*-$[(Im)_2Cl_4Ru]$ (ICR), yields the anionic species, *trans*-$[(Im)_2Cl_4Ru]^-$, whose mechanism of action appeared to be transferrin-mediated.[18,19,21]

### Materials and Methods

**Materials.** *cis*-$[Ru(Cl_2(NH_3)_4]Cl$ and *trans*-(ImH)$[Ru(Im)_2Cl_4]$ (ImH = imidazolium ion) were prepared by literature methods.[20,22] Human serum apotransferrin was obtained from Sigma Biochemical. Specialty gases (10% ±0.4 % $O_2$, 5% ± 0.4%, $CO_2$ balance $N_2$ and 5%±0.4% $O_2$, 5%±0.4% $CO_2$ balance $N_2$ by GC analysis) were obtained from Wesco. Buffer A consists of: 66 µL of 4.5 M $MgCl_2$, 1.24 g of Tris-HCl buffer, and 150 µL of 2-mercaptoethanol, diluted to one liter and adjusted to pH 7.4.

**Cell Culture.** The HeLa cell culture was a gift from the laboratory of A.T. Annunziato (Dept. Biology, Boston College). Cells were maintained in standard media: SMEM (BioWhittaker), supplemented with 1% v/v l-glutamine, 0.5% v/v penicillin-streptomycin, 10% v/v fetal bovine serum, and 25 µM Hepes buffer in an incubator at 37 C, with stirring. The culture was maintained at a concentration of 3 x $10^5$ cells/mL as determined by counting on a counting slide. Millipore purified water was used in all dilutions and purifications involving cells and cellular materials.

Control and experimental cultures were begun at the concentration of 3 x $10^5$ cells/mL with a predetermined volume of ruthenium drug stock solution added through a sterile-filtering (0.45 μm) syringe to give the desired [Ru] in the final cell volume (50 mL). After 24 h, the cultures were counted and the cell counts expressed as per cent growth relative to the control ($\%G_r = 100 \times N_{Ru}/N_{control}$), where $N_{control}$ is the cell density in the control and $N_{Ru}$ is the cell density in the Ru-added culture, or per cent inhibition ($\%IG_r = 100 - \%G_r$). Controls (no Ru added) involved a stock culture begun at 3 x $10^5$ cells/mL and incubated for 24 h under the same conditions as the Ru-added cultures. Controls and Ru-added cultures were run for each type of experiment in which the partial pressure of oxygen and concentration of transferrin were varied. Cultures were acclimated to the reduced oxygen conditions for 24 h prior to use with the ruthenium complexes.

**Ru-DNA Measurements.** In order to extract the nuclear DNA, cultures were spun down at approximately 1000 rpm for 10 minutes in an International Equipment Company HN-S II centrifuge. The supernatant media was decanted from the cell pellet and the cell pellet was resuspended and washed three times with buffer A to assure complete removal of the Ru-containing media. The cell pellet was resuspended with buffer A, and lysed with a glass homogenizer. A pellet of cell nuclei was isolated by centrifugation at approximately 2500 rpm (10 min.) and was resuspended, centrifuged and washed three times with buffer A. Nuclei kept for further use were maintained in an ice bath or frozen overnight (-20 C) in buffer A.

Nuclear membranes were lysed by a protease K solution (50 μM of 10% SDS, 20 μM 10% protease K solution (Sigma) and 935 μM of isolated nuclei). This solution was placed in 1.5 mL Eppendorf tubes and left to digest for 24 h at 37 C. The DNA was isolated by extraction with an equivalent volume of 1:1 phenol:chloroform. Phenol:chloroform. extractions were continued until there was no white, stringy material visible at the phase interface. The solution was then extracted with an equivalent volume of molecular biology grade chloroform, until there were no white filaments visible at the phase interface. The aqueous phase was removed, and the DNA precipitated by the addition of 0.2 M sodium chloride in 70 % ethanol. This solution was placed in a -78 C ice bath, and centrifuged in a Fisher microfuge for 15 minutes. The DNA pellet was retrieved by decanting off the ethanol, and resuspending in another 1 mL of NaCl/ethanol solution. This DNA precipitation process was repeated twice more to be sure no free Ru was carried through with the DNA. The DNA was quantified by UV absorption at 260 nm ($\varepsilon = 6600\ M^{-1}\ cm^{-1}$ per phosphate) in a Tris-EDTA buffer at pH 7.8. Ruthenium was quantified by graphite-furnace atomic absorption (Perkin Elmer) using the temperature program [time (s) and T (C)]: 4, 70; 5, 200; 4, 800; 4, 1500; 1, 2800; 2, 2800. Results are reported as normalized per DNA nucleotide, i.e. as the ratio of Ru bound to the DNA per DNA phosphate, $Ru_{DNA}/P_{DNA}$.

### Results

In order to determine whether the ruthenium in the nuclear DNA samples was tightly bound, several samples of DNA extracted from HeLa cells that had been incubated with ICR and CCR were subjected to exhaustive dialysis (3 x in 0.010 M HEPES/0.010 M $NaHCO_3$) and requantified. The relative changes in $Ru_{DNA}/P_{DNA}$ for each drug following dialysis were *not* consistently lower than the original mean, but were randomly distributed with relative standard deviations of ± 11% for CCR and ± 8% for ICR, which is consistent with both drugs binding tightly to the nuclear DNA.

The effect of hypoxia on cell toxicity and DNA binding was studied by running cultures at one half and one quarter the partial pressure of oxygen present in the atmosphere. Toxicity and nuclear DNA binding of CCR and ICR in HeLa cell cultures at the various partial pressures of oxygen ($P_{O_2}$) are presented in Tables I and II and Figures 1 - 6. For purposes of comparison, approximately linear ranges of the variables were treated by standard linear least squares methods. For all $P_{O_2}$'s, $\%IG_r$ versus log[Ru] plots for both the CCR the ICR complexes show a straightforward dose-response correlation (see Figs. 1, 3 and 5). The corresponding $IC_{50}$'s listed in Table I indicate increased toxicity of the ruthenium agents as $P_{O_2}$ decreases. Nuclear DNA binding by ruthenium, which is expressed as ruthenium atoms per 1000 DNA nucleotides (see $Ru_{DNA}/10^3P_{DNA}$ values in Table I), increased linearly with [Ru] (see Figures 2, 4 and 6) and correlated with cell toxicity at any given $P_{O_2}$. When expressed at a constant ruthenium concentration (10 μM), DNA binding increases with decreasing $P_{O_2}$; whereas at [Ru] = $IC_{50}$, Ru-DNA binding remains relatively constant over the varying oxygen pressures (Table I).

The effect of transferrin on cell toxicity and DNA binding of the ruthenium complexes was studied by running cultures with added apotransferrin ($[Tf]_a$) under normal atmospheric conditions. Transferrin in the media itself is ~3.3 μM. As added apotransferrin enhances cell growth at low concentrations, but inhibits cell growth at $[Tf]_a$ > 0.7 μM (50 μg/mL), the effect of apotransferrin was studied over the range 0 - 0.7 μM (see Figure 8). At constant [Ru] = 10 μM, increasing $[Tf]_a$ resulted in increasing inhibition of cell growth for both compounds. At the $IC_{50}$ values for $[Tf]_a$ under these conditions, ruthenium binding to DNA was approximately twice that when no added transferrin was present (cf. $Ru_{DNA}/10^3P_{DNA}$ values in Tables I and II).

**Table I.** Concentrations of ruthenium complexes sufficient to inhibit cell growth to 50% of that of the control ($IC_{50}$), the amount of Ru bound to DNA per thousand nucleotides at [Ru] = $IC_{50}$, and the amount of Ru bound to DNA per thousand nucleotides at constant [Ru] (10 μM) at $P_{O2}$ = 159, 76, and 38 torr. ICR = [ImH]*trans*-$[(Im)_2Cl_4Ru]$. CCR = *cis*-$[Cl_2(NH_3)_4Ru]Cl$.

| Compound | $P_{O2}$ (torr) | $IC_{50}$ (μM) | $(Ru_{DNA}/P_{DNA}) \times 10^3$ at [Ru] = $IC_{50}$ | $(Ru_{DNA}/P_{DNA}) \times 10^3$ at [Ru] = 10 μM |
|---|---|---|---|---|
| ICR | 159 | 2.0 ± 0.3 | 0.20 ± 0.04 | 0.36 ± 0.07 |
| CCR | 159 | 3.5 ± 0.4 | 0.10 ± 0.02 | 0.27 ± 0.06 |
| ICR | 76 | 1.0 ± 0.1 | 0.63 ± 0.26 | 1.4 ± 0.6 |
| CCR | 76 | 1.7 ± 0.5 | 0.44 ± 0.06 | 1.1 ± 0.2 |
| ICR | 38 | 0.006 ± 0.003 | 0.48 ± 0.16 | 4.0 ± 1.3 |
| CCR | 38 | 0.023 ± 0.007 | 0.37 ± 0.08 | 1.7 ± 0.4 |

**Table II.** Concentrations of added apotransferrin ($[Tf]_a$) sufficient to inhibit cell growth to 50% of that of the control ($IC_{50}$) at [Ru] = 10 μM and $P_{O2}$ = 159 torr.

| Compound | $IC_{50}$ (μM) | $(Ru_{DNA}/P_{DNA}) \times 10^3$ at [Ru] = 10 μM, $[Tf]_a$ = $IC_{50}$, |
|---|---|---|
| **ICR** | 0.14 ± 0.03 | 0.6 ± 0.2 |
| **CCR** | 0.25 ± 0.05 | 0.7 ± 0.4 |

**Figure 1.** Inhibition of HeLa cell growth versus [Ru] (μM) (logarithmic scale) for ruthenium complexes at $P_{O_2}$ = 159 torr . Lines are logarithmic linear least squares fits. Circle points, solid line, *cis*-$[Cl_2(NH_3)_4Ru]Cl$ (CCR); square points, dashed line, [ImH]*trans*-$[(Im)_2Cl_4Ru]$ (ICR).

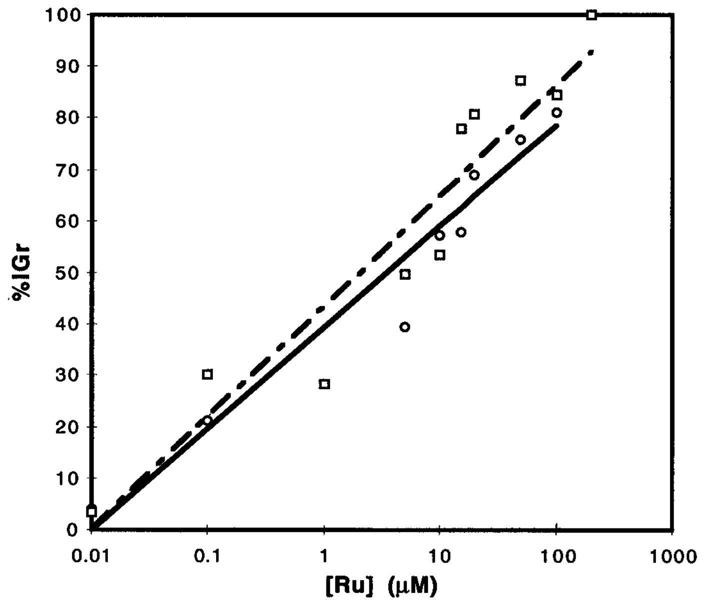

**Figure 2.** Binding of Ru to nuclear DNA versus culture [Ru] (μM) for ruthenium complexes at $P_{O_2}$ = 159 torr . Lines are linear least squares fits. Circle points, solid line, *cis*-$[Cl_2(NH_3)_4Ru]Cl$ (CCR); square points, dashed line, [ImH]*trans*-$[(Im)_2Cl_4Ru]$ (ICR).

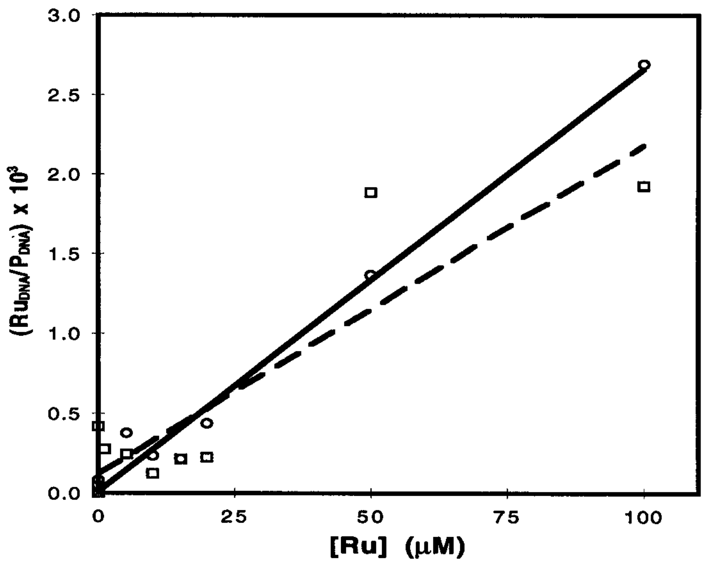

**Figure 3.** Inhibition of HeLa cell growth versus log[Ru] (μM) for ruthenium complexes at $P_{O_2}$ = 76 torr . Lines are logarithmic linear least squares fits. Circle points, solid line, *cis*-$[Cl_2(NH_3)_4Ru]Cl$ (CCR); square points, dashed line, [ImH]*trans*-$[(Im)_2Cl_4Ru]$ (ICR).

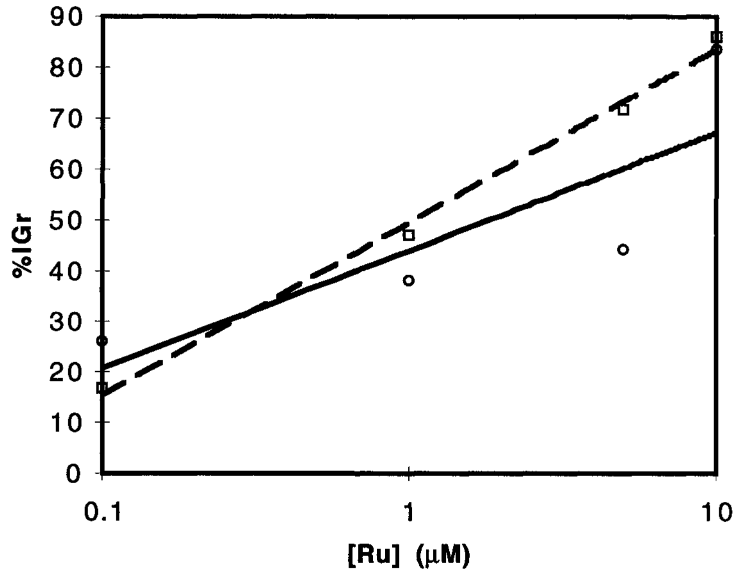

**Figure 4.** Binding of Ru to nuclear DNA versus culture [Ru] (μM) for ruthenium complexes at $P_{O_2}$ = 76 torr . Lines are linear least squares fits. Circle points, solid line, *cis*-$[Cl_2(NH_3)_4Ru]Cl$ (CCR); square points, dashed line, [ImH]*trans*-$[(Im)_2Cl_4Ru]$ (ICR).

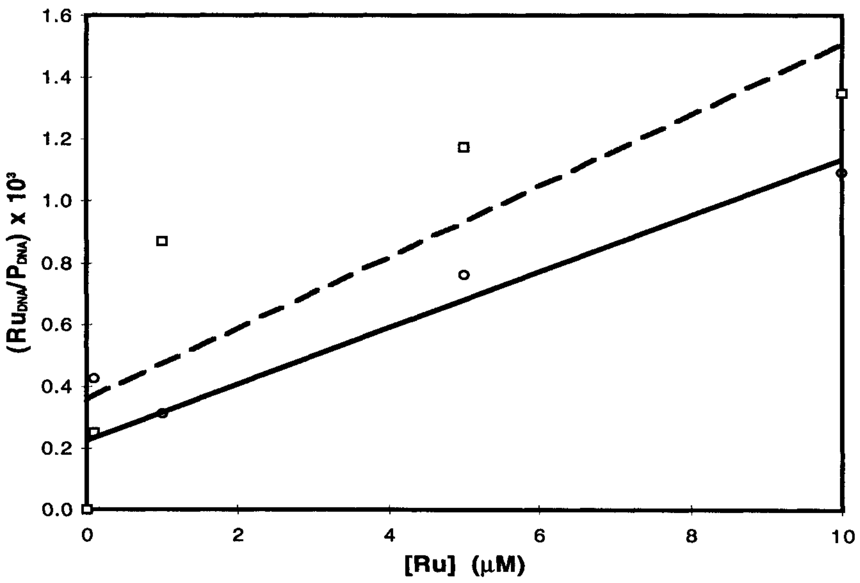

**Figure 5.** Inhibition of HeLa cell growth versus log[Ru] (μM) for ruthenium complexes at $P_{O_2}$ = 38 torr . Lines are logarithmic linear least squares fits. Circle points, solid line, *cis*-$[Cl_2(NH_3)_4Ru]Cl$ (CCR); square points, dashed line, [ImH]*trans*-$[(Im)_2Cl_4Ru]$ (ICR).

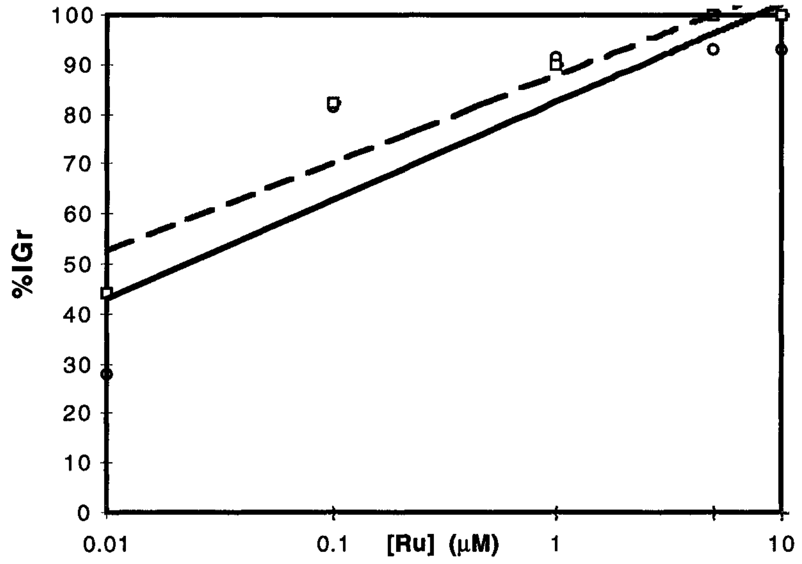

**Figure 6.** Binding of Ru to nuclear DNA versus culture [Ru] (μM) for ruthenium complexes at $P_{O_2}$ = 38 torr . Lines are linear least squares fits. Circle points, solid line, *cis*-$[Cl_2(NH_3)_4Ru]Cl$ (CCR); square points, dashed line, [ImH]*trans*-$[(Im)_2Cl_4Ru]$ (ICR).

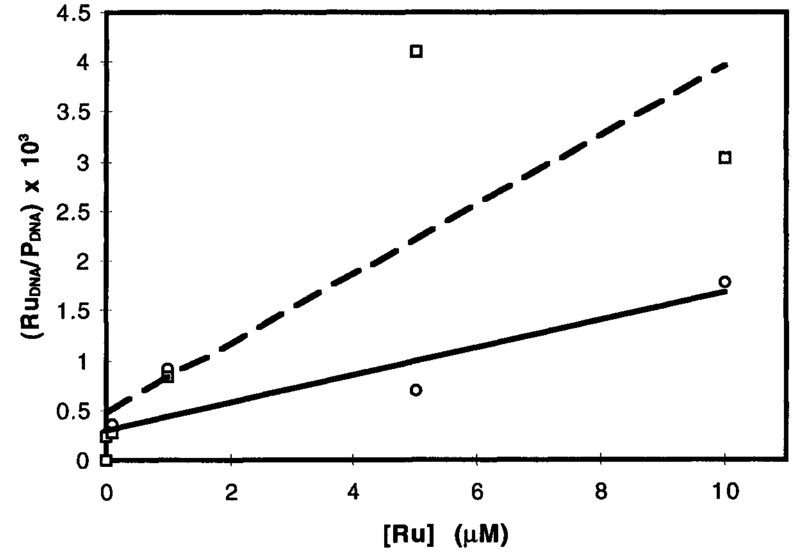

**Figure 7**. Correlation between toxicity ($IC_{50}$ values) and $\log[P_{O_2}]$. Lines are linear least squares fits. Circle points, solid line, *cis*-$[Cl_2(NH_3)_4Ru]Cl$ (CCR); square points, dashed line, [ImH]*trans*-$[(Im)_2Cl_4Ru]$ (ICR).

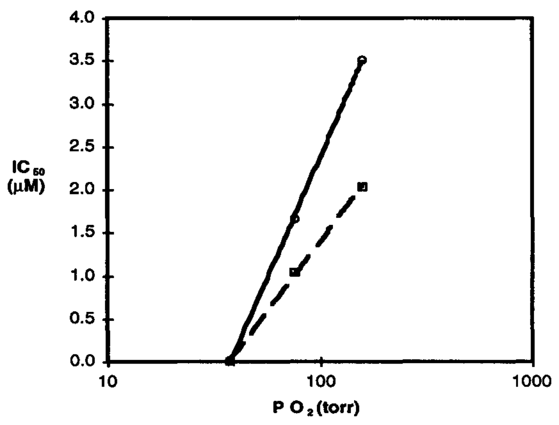

**Figure 8**. Inhibition of HeLa cell growth versus added transferrin, $[Tf]_a$ (μM), for cultures with [Ru] = 10 μM of CCR or ICR at $P_{O_2}$ = 159 torr compared to the relative growth with no Ru present. The growth scale (%Gr) for the diamond points (top alternately dashed line, no Ru present is relative to a control at [Ru] = 0. This line is fit by a 2-point running average. Circle points, solid line, *cis*-$[Cl_2(NH_3)_4Ru]Cl$ (CCR); square points, dashed line, [ImH]*trans*-$[(Im)_2Cl_4Ru]$ (ICR) are for added $[Tf]_a$ at [Ru] = 10 μM of CCR or ICR relative to the control for the respective complex at the same [Ru]. These lines were fitted by a least squares fit to a decreasing exponential equation. Growth in the [Ru] = 10 μM controls relative to [CCR] = 0 or [ICR] = 0 can be estimated from Figure 1.

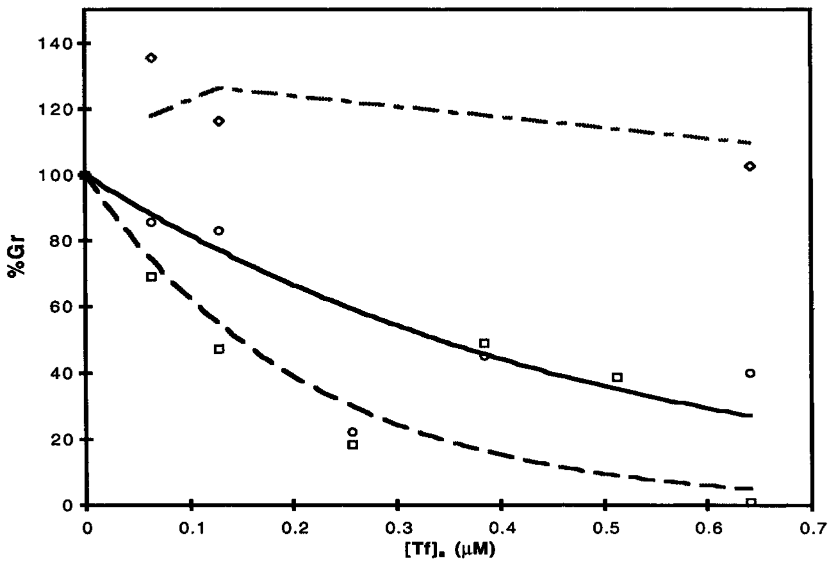

### Discussion

Despite their differences in charge, the two complexes behaved remarkably alike throughout this study. Both complexes appear to access nuclear DNA quite well at therapeutically reasonable concentrations and the cytotoxicity of both is enhanced by added transferrin and reduced oxygen. ICR is somewhat more toxic than CCR under all conditions. Plots of $IC_{50}$ versus $\log[P_{O2}]$ and $Ru_{DNA}/10^3P_{DNA}$ at [Ru] = 10 µM versus $\log[P_{O2}]$ (Figure 7, see Table I) are linear indicating increased toxicity through increased DNA binding at lower oxygen tensions for both complexes. While the effect of transferrin is higher for ICR, both drugs exhibit significantly increased toxicity relative to the controls as the apotransferrin concentration increases (Figure 8). As the data were treated by linear methods, the positive intercepts on many of the DNA binding curves (Figs. 2, 4 and 6) are artifacts of the statistical treatment, but are also consistent with an active transport mechanism providing entry for these complexes into the HeLa cells.

As both complexes are firmly attached to the nuclear DNA, covalent binding appears to be involved. Coordination by most ruthenium(II) and ruthenium(III) complexes occurs at the N7 of purine bases (usually guanine),[23] but binding to the exocyclic amines of adenine and cytosine is also possible,[24] as is coordination across the double bond in uracils.[25] Since both ICR and CCR have at least two substitutable sites, DNA cross linking may be possible.

Consistent with the hypothesis that the antitumor activity of these complexes is dependent upon DNA binding, the level of cytotoxicity (%IGr) and DNA binding ($Ru_{DNA}/P_{DNA}$) correlate linearly (See Figures 1-6). The $Ru_{DNA}/P_{DNA}$ values at the $IC_{50}$ concentrations of the complexes remain relatively constant at all partial pressures of oxygen, indicating that similar levels of DNA binding are similarly toxic at all oxygen tensions (Table I). In harmony with the activation-by-reduction hypothesis for both complexes, both the inhibition of cell growth and DNA binding increase at constant [Ru] as $P_{O2}$ decreases. In particular, at the lowest $P_{O2}$ (38 torr), the cytotoxicity of both complexes is greatly enhanced (Table I).

While the reduction potential at neutral pH (E°' = -262 mV)[26,27] of ICR is within a biologically accessible range, significant reduction is not expected. However, reduction of ICR is pH dependent and becomes more favored at lower pH.[28] At pH 5, which results when protons are pumped into the endosomes to release $Fe^{III}$ (and presumably $Ru^{III}$) from the Fe-transferrin-receptor complex,[19,29] the reduction potential for ICR is ~-100 mV. Reduction should also be facilitated by loss of chloride ($t_{1/2}$ = 3.5 h at 37 C),[28] which should be substantial over the 24 h experiments presented here. Since dissociation of $Cl^-$ precedes or is concomitant with transferrin uptake,[21] reduction of some metabolic forms of ICR would appear likely.

As Sadler has pointed out, the anomalous pH behavior in the electrochemistry of ICR[28] is reminiscent of that seen in the electrochemistry of $[L(NH_3)_5Os^{III,II}]$ (L = imidazole ring ligand),[30] in which the d-orbitals are also expanded, but owing to a higher atomic number rather than anionic charge. It may be that imidazoles by virtue of their ability to serve as $\pi \rightarrow d_{\pi}$-donors or $d_{\pi} \rightarrow \pi$-acceptors (depending on the energy and extension of the d-orbitals)[27] help to provide a polarizable site for proton addition, which raises the reduction potential of the metal ion.

As the concentration of apotransferrin increases (with no Ru), cell growth initially increases and then gradually decreases, so that at added concentrations greater than 50 µg/mL, transferrin itself is toxic. When the effect of added apotransferrin was studied within the range of approximately normal cell growth at [Ru] = 10 µM, growth inhibition increased as the apotransferrin concentration increased. Since transferrin is known to bind ICR, such behavior for this complex is not surprising. The observation of similar behavior for CCR, suggests that CCR also enters cancer cells by a transferrin-mediated route and that this is comparable in efficiency to that for ICR.

The similarity in transferrin effects may be due to the comparable aquation rates of the two complexes. At 37 °C CCR substitutes water for chloride at a rate of $3.5 \times 10^{-5}$ $s^{-1}$ ($t_{1/2}$ = 5.5 h),[31] while that for ICR is $5.3 \times 10^{-5}$ $s^{-1}$ ($t_{1/2}$ = 3.7 h).[28] Considering that cellular uptake and transfer into the cell nucleus precedes DNA binding, these substitution rates seem slow in accounting for the amount of ruthenium binding to the intracellular DNA. More rapid substitution is consistent with an activation-by-reduction pathway.

### Conclusion

Both cationic (CCR) and anionic (ICR) antitumor complexes behaved remarkably similarly, suggesting analogous mechanisms of action, which probably involves transferrin binding to actively transport the Ru into the cancer cell, possibly followed by intracellular activation by reduction to facilitate DNA binding. On average for the various oxygen tensions and for both complexes, $IC_{50}$'s occur when one ruthenium is coordinated per 1000-2000 DNA base pairs.

### Acknowledgment

This work was supported by NIH Grant GM26390 and a Research Incentive Grant from Boston College.

### References

(1) M. J. Clarke, *Met. Ions Biol. Syst.,* 1980, **11**, 231-283.

(2) A. D. Kelman, M. J. Clarke, S. D. Edmonds, H. J. Peresie, *J. Clin. Hematol. Oncol.,* 1977, **7**, 274-288.

(3) R. E. Yasbin, C. R. Matthews, M. J. Clarke, *Chemico-Biol. Interact.,* 1980, **30**, 355.

(4) K. A. Marx, R. Kruger, M. J. Clarke, *Molec. Cell. Biochem.,* 1989, **86**, 155-162.

(5) K. A. Marx, C. Seery, P. Malloy, *Mol. Cell. Biochem.,* 1989, **90**, 37-95.

(6) J. R. Durig, J. Danneman, W. D. Behnke, E. E. Mercer, *Chem.-Biol. Interact.,* 1976, **13**, 287-294.

(7) B. K. Keppler, W. Rupp, U. W. Juhl, H. Endres, R. Niebl, W. Balzer, *Inorg. Chem.,* 1987, **26**, 4366-4370.

(8) M. H. Seelig, M. R. Berger, B. K. Keppler, *J. Cancer Res. Clin. Oncol.,* 1992, **118**, 195.

(9) M. R. Berger, F. T. Garzon, D. Schmal, B. K. Keppler, *Anticancer Res.,* 1989, **9**, 761-5.

(10) B. K. Keppler, M. Henn, U. M. Juhl, M. R. Berger, R. Niebel, F. E. Wagner in *Ruthenium and Other Non-Platinum Metal Complexes in Cancer Chemotherapy,* eds. M. J. Clarke, Springer-Verlag, Heidelberg, edn., 1989; Vol. 14, pp 41-70.

(11) M. J. Clarke, M. Stubbs, *Met. Ions in Biol. Syst.,* 1996, **32**, 727-780.

(12) D. Miklavcic, G. Sersa, S. Novakovic, *J. Bioelect.,* 1990, **9**, 133.

(13) P. Okunieff, E. P. Dunphy, P. Vaupel, *Adv. Exp. Med. Biol.,* 1994, **345**, 485.

(14) P. Vaupel, K. Schlenger, C. Knoop, *Canc. Res.,* 1991, **51**, 3316.

(15) S. C. Srivastava, P. Richards, G. E. Meinken, P. Som, H. L. Atkins, S. M. Larson, A. Grunbaum, J. S. Rasey, M. G. Dowling, M. J. Clarke in *Radiopharmaceuticals II,* eds. J. A. Sorensen, Society of Nuclear Medicine, New York, edn., 1979; Vol. 265-269.

(16) P. Som, Z. H. Oster, K. Matsui, G. Gugliemi, B. Persson, M. L. Pellettieri, S. C. Srivastava, P. Richards, H. L. Atkins, A. B. Brill, *Eur. J. Nucl. Med.,* 1983, **8**, 491.

(17) S. C. Srivastava, L. F. Mausner, M. J. Clarke in *Ruthenium and other Non-Platinum Metal Complexes in Cancer Chemotherapy,* eds. M. J. Clarke, Springer-Verlag, Heidleberg, edn., 1989; Vol. 10, pp 111-150.

(18) F. Kratz, B. K. Keppler, L. Messori, C. Smith, E. N. Baker, *Metal-Based Drugs,* 1994, **1**, 169-173.

(19) F. Kratz, M. Hartmann, B. Keppler, L. Messori, *J. Biol. Chem.,* 1994, **269**, 2581-2588.

(20) B. K. Keppler, D. Wehe, H. Endres, W. Rupp, *Inorg. Chem.,* 1987, **26**, 844-846.

(21) L. Messori, F. Kratz, E. Alessio, *Metal-Based Drugs,* 1996, **3**, 1-9.

(23) M. J. Clarke, H. Taube, J. Am. Chem. Soc., 1974, 96, 5413-5419.

(24) M. J. Clarke, J. Am. Chem. Soc., 1978, 100, 5068-5075.

(25) R. E. Shepherd, S. Zhang, F.-T. Lin, R. A. Kortes, Inorg. Chem., 1992, 31, 1457.

(26) E. Alessio, G. Balducci, A. Lutman, G. Mestroni, M. Calligaris, W. M. Attia, Inorg. Chim. Acta, 1993, 203, 205-217.

(27) M. Clarke, V. Bailey, P. Doan, C. Hiller, K. J. LaChance-Galang, H. Daghlian, S. Mandal, C. Bastos, D. Lang, Inorg. Chem., 1996, 35, in press.

(28) O. M. N. Dhubhghaill, W. Hagen, B. K. Keppler, L. K-G., J. Sadler, J. Chem. Soc. Dalt. Trans., 1994, 3305-3311.

(29) S. J. Lippard, J. M. Berg, Principles of Bioinorganic Chemistry, University Science, Mill Valley, CA, 1994, Vol. .

(30) A. Johnson, L. A. O'Connell, M. J. Clarke, Inorg. Chim. Acta, 1993, 210, 151-157.

(31) A. Broomhead, L. Kane-Maguire, Inorg. Chem., 1968, 7, 2519-2523

**Received: June 25, 1996 - Accepted: July 22, 1996 -**
**Received in revised camera-ready format: September 19, 1996**

